# Large inter-stock differences in catch size-at-age of mature Atlantic salmon observed by using genetic individual origin assignment from catch data

**DOI:** 10.1371/journal.pone.0247435

**Published:** 2021-04-06

**Authors:** Marja-Liisa Koljonen, Michele Masuda, Irma Kallio-Nyberg, Jarmo Koskiniemi, Irma Saloniemi

**Affiliations:** 1 Natural Resources Institute Finland (Luke), Production Systems, Animal Genetics, Helsinki, Finland; 2 Auke Bay Laboratories, Alaska Fisheries Science Center, National Marine Fisheries Service, National Oceanic and Atmospheric Administration, Juneau, Alaska, United States of America; 3 Natural Resources Institute Finland (Luke), Ecosystems and Ecology, Fish Stocks and Environment, Helsinki, Finland; 4 Department of Agricultural Sciences, University of Helsinki, Helsinki, Finland; 5 Department of Biology, University of Turku, Turku, Finland; University of Iceland, ICELAND

## Abstract

Genetic individual assignment of river stock of origin of mixed stock catch fish offers a tool to analyze size differences among river stocks. Data on the genetically identified river stock of origin of individual fish from commercial mixed stock catches were used to compare the catch size-at-age of mature Atlantic salmon catch fish (*Salmo salar*) from different rivers in the Baltic Sea. In this application of genetic mixed stock modeling, individual assignments of the river stock of origin were analyzed together with length- and weight-at-age data for individual catch fish. The use of four genetic stock identification based methods was compared for defining the length distributions of caught mature salmon in different river stocks. The catch data included information on maturing salmon in the northern Baltic Sea over the years 2000–2013. DNA microsatellite data on 17 loci and information on the smoltification age were used to assign spawners to their stock of origin. All of the compared methods for using probabilistic stock of origin data in our case yielded very similar estimates of the final mean length distributions of the stocks. The Bayesian mixture model yielded slightly more conservative estimates than the direct probability method, threshold method, or the modified probability method. The catch size between spawners of a same sex and age from river stocks differed significantly and the differences were large. The mean catch weight of 1-sea-winter old mature males in different rivers varied from 1.9 kg to 2.9 kg, from 5.1 kg to 7.5 kg for 2-sea-winter old males, from 5.0 kg to 7.2 kg for 2-sea-winter old females, and from 8.2 kg to 10.8 kg for 3-sea-winter-old females. The mean size of caught wild salmon spawners in each year-class was on average smaller than that of the hatchery-reared and sea ranched stocks.

## Introduction

Growth rate, age at maturity, and the size of spawners are important factors for the survival and fitness of Atlantic salmon (*Salmo salar*) populations [1–3] because growth rate is tightly linked to the maturation process and the size of spawners and to the fecundity of populations [4]. Atlantic salmon inherit rather than acquire a tendency to mature at a certain age [5], and a large part of maturation age variation is linked to one gene, with some polygenic influence as well [6,7]. The size of the spawning stock is also economically important, as the final catch weight of mature salmon varies considerably, from less than 2 kg to over 10 kg, mainly because of spawning age differences. The large majority of Atlantic salmon in the Baltic Sea spawn only once, and the maximum size these fish reach in their lifetime is thus their size at the first individual spawning age, usually after 1 to 3 sea years/winters (SW) in the sea on their feeding migration [8,9].

Little is known about the growth differences between naturally reproducing Atlantic salmon stocks of different rivers, especially in the Baltic Sea area. An extensive comparison of the growth of 23 Norwegian Atlantic salmon stocks was conducted by Holm and Naevdal [10]. They did controlled breeding experiments for several generations and assessed heritability of growth at different life stages. They also reported wide variation in growth and age at maturation between Atlantic salmon populations of different rivers.

The monitoring of changes in the growth and size of spawners is important for identifying the effects of environmental selective forces, such as climate change or fisheries, as a decrease in the size of spawners may also reduce the fecundity and reproduction of the stocks [11–13]. Differences among wild stocks are especially interesting, as they potentially reflect local adaptive differences. The monitoring of growth trends in hatchery-reared and sea-ranched stocks is also essential for the quality control of hatchery breeding and rearing processes[14]. In addition, there may be unknown inherited growth differences among hatchery stocks, which are usually difficult to identify without extensive and expensive tagging and releasing programs or organized experiments in the hatchery environment [15]. Stock-specific marine growth studies on mature salmon in the Baltic Sea were first reported in the 1930s and 1940s, when they were based on catch samples from the wild spawning stocks in the rivers [16,17], and more recently on externally tagged fish to identify their river of origin from catches [18].

The offshore catch sample analyzed here allowed much larger sample sizes than would have been obtainable by sampling salmon in their home rivers. Instead of tagging, information on the stock of origin of individual salmon was based on probabilistic individual assignment (IA) data derived from Bayesian genetic stock identification (GSI), which is a form of mixed stock analysis (MSA). This method also enabled the analysis of wild stocks, which are rarely tagged to any large extent. Our data came from commercial fisheries which tells about fish size in catches and also probably reflects the differences in the breeding populations as well, in most cases.

The stock and stock group proportions of Baltic Sea Atlantic salmon catches have been analyzed annually for stock assessment purposes as part of an EU sampling program. Data on the assigned origin of individual catch fish together with their quantitative traits were additionally utilized in the current analysis. This has, to our knowledge, rarely been carried out, partly because the probabilistic nature of information on the stock of origin of the fish creates a challenge that needs to be addressed [19,20]. Genetic individual assignment of the river stock of origin has previously been used to classify individual Atlantic salmon according to their source as if this source was known 100% correctly [21,22]. Even if accepted probability thresholds are used, the information above the chosen threshold is considered to be 100% correct for those individuals. However, methods are available that take into account the uncertainty in individual assignments.

Here we first compared four methods of identifying stock of origin and corresponding size differences among contributing stocks on a subset of the total catch data. Second, we applied one of the methods in defining size differences among contributing river stocks on the total catch data for each sex and age group. In the method comparison we tested four methods of using GSI information on the same length data for 2 SW (sea-winter) mature Atlantic salmon females, which comprised the largest age group. The compared methods were

1. the direct use of individual assignment probabilities (IAPs) as weights for the value of the trait of interest (= direct probability method, DPM),
2. the traditional threshold method using two criteria, 0.59 and 0.8, in which only individuals with higher IAPs than the set threshold were included in the size analysis (= threshold method, THM),
3. a modification of the probability method, called the reweighted method (RWM), in which only a share of the individual assignment probability (IAP) for the stock of origin that exceeded the level of the probability for the other stocks was used for that individual, and also
4. the Bayesian mixture model (BMM) [20,23], in which the growth distribution of the river stocks was directly estimated in the model.

For methods 1, 2, and 3, SAS mixed models were used to define the size distributions based on length and weight.

This study had two steps and goals: 1) to compare methods for assessing the mean lengths of salmon stocks from genetic mixed stock data and 2) to apply one of the methods to assess average size-at-age differences in catches among spawners of a total of 14 wild and sea-ranched Atlantic salmon stocks over the years 2000 to 2013, excluding 2001.

## Material and methods

### Offshore sampling of catch fish scales and DNA-analysis

Scale samples of catch salmon were collected by fishermen from the commercial coastal trap net and driftnet fisheries [24]. The annual salmon catch scale data collection is part of the EU sampling program National Data Collection Programme under Council Regulation (EC) N° 199/2008, which is done for salmon fisheries management and catch quota decision. Scales from caught salmon are systematically collected for fish age determination, dried and archived. The catch length (total length), weight, and sex of the individual catch salmon were recorded, as well as the catch date and site by fishermen. The age at smoltification, sea age, and DNA microsatellite multilocus genotypes were analyzed from the sampled scales. Sex was not recorded for all individuals, which reduced the sample sizes. Scales for DNA-analysis were resampled from the wider catch scale collection. The smolt-age information on catch fish was used as one variable in GSI, in addition to 17 DNA microsatellite loci, and their sea-age was used to classify spawners according to age.

**Fig 1:**
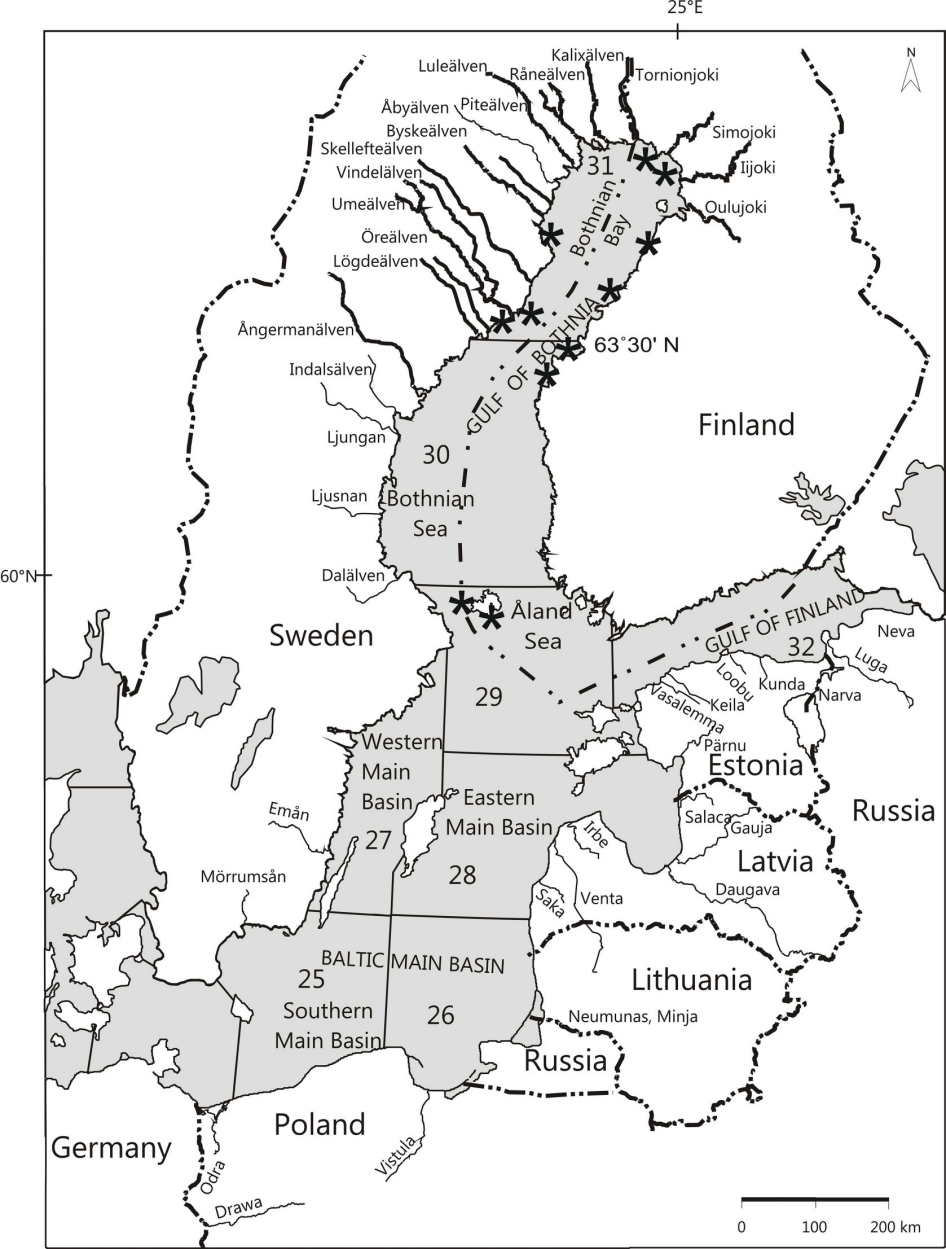
Map of the Baltic Sea, with the rivers of origin of the studied Atlantic salmon stocks in bold and the sites of the salmon catch samples indicated with asterisks (*) in Finnish and Swedish coastal waters, in the Åland Sea, Bothnian Sea, and Bothnian Bay areas. The figure is edited and only for illustrative purposes.

To target the DNA sampling on mature fish, catch salmon scales were resampled from scale archive collections taken from fish caught on their spawning migration during May, June, and July in 2000 and in the years 2002–2013. The mean catch month for salmon in all stocks was June, the month when mature spawners return to the river mouths. Nearly all (99.9%) of the sampled fish of this study were first-time spawners with ages ranging from 1 to 5 sea-winters (SW), and sizes from 50 cm to 120 cm. As most of the fish were aged from 1 to 3 SW, growth was only analyzed in these three age classes. The catches were from three Baltic Sea sub-basins (Fig 1) along the coast of the Gulf of Bothnia (GB): the Åland Sea (ICES sea subdivision 29; samples mainly from the first week of June), Bothnian Sea (BS; ICES sea subdivision 30; mainly from the third week of June), and Bothnian Bay (BB; ICES sea subdivision 31; mainly from the fourth week of June) (Fig 1) [19]. Salmon stop feeding and growing when they begin their spawning migration, so the size at maturation is their final size if they spawn only once. Fish from all sites represent the same annually northward migrating group of mature salmon. Fish samples from all catch sites were pooled in the analysis (Fig 1).

To protect immature salmon, the legal minimum landing size for salmon in the Baltic Sea is 60 cm, except in the Bothnian Bay, north of latitude 63°30’ N (Fig 1), where it has been 50 cm since 1993. This regulation enabled us to sample small, less than 60 cm, 1 SW mature males which are quite common in populations [8]. The river mouths of all studied salmon rivers, expect Ångermanälven, are north of this latitude. Sample sizes for this river were small and no 1 SW males were included in the sample.

All catch salmon were assigned to their river stock of origin. From this data set, individual fish assigned to the most abundant eight wild and six hatchery stocks were initially selected for the growth analysis, since they potentially provided sufficient numbers for comparisons, when having at least 40 individuals assigned to that river stock. The eight wild salmon stocks were from the rivers Tornionjoki, Kalixälven, Byskeälven, Vindelälven Åbyälven, Öreälven, Lögdeälven, and Simojoki. The six hatchery-reared stocks were Tornionjoki hatchery stocks and also hatchery stocks from the rivers Iijoki, Oulujoki, Luleälven, Skellefteälven, and Ångermanälven (Fig 1, Table 1).

**Table 1:** Mean probability of the stock of origin for the individually assigned catch fish from the Atlantic salmon offshore catches in the Bothnian Bay during 2000–2013.

	Stock, wild (W)	n	n	Mean P	n	Mean P	Sex	% of	1-2year	smolts
	or hatchery (H)	Total	P>0.50	P>0.50	P>0.59	P>0.59	ratio	2.5%	Med.	97.5 %
1	Tornionjoki, W	1524	1416	0.79±0.15	1207	0.83±0.12	0.43	4.3	5.6	7.3
2	Kalixälven, W	886	817	0.77±0.15	664	0.81±0.11	0.41	3.9	5.7	8.0
3	Byskeälven, W	500	456	0.84±0.15	414	0.87±0.12	0.42	22.5	30.2	39.4
4	Vindelälven, W	185	174	0.91±0.13	168	0.92±0.10	0.46	31.1	36.9	43.8
5	Åbyälven, W	138	109	0.73±0.13	86	0.78±0.13	0.40	22.0	29.9	39.6
6	Öreälven, W	43	34	0.84±0.18	31	0.87±0.15	0.15	14.4	21.4	29.7
7	Lögdeälven, W	225	221	0.95±0.10	217	0.96±0.08	0.29	21.6	29.3	38.5
8	Simojoki, W	197	159	0.78±0.17	128	0.85±0.13	0.58	42.3	52.2	61.7
** **	**Total wild**	**3698**	**3386**	** **	**2915**	** **	** **	** **	** **	** **
9	Tornionjoki, H	623	519	0.79±0.15	440	0.83±0.13	0.60	99.8	100.0	100.0
10	Iijoki, H	482	428	0.78±0.16	381	0.88±0.12	0.69	99.8	100.0	100.0
11	Oulujoki, H	466	433	0.91±0.14	410	0.93±0.12	0.62	99.8	100.0	100.0
12	Luleälven, H	204	178	0.83±0.15	160	0.86±0.12	0.54	99.8	100.0	100.0
13	Skellefteälven, H	111	100	0.85±0.15	92	0.87±0.13	0.58	99.8	100.0	100.0
14	Ångermanälven,H	51	42	0.85±0.14	40	0.86±0.13	0.59	99.8	100.0	100.0
	**Total reared**	**1937**	**1700**	** **	**1523**					
** **	**Overall total**	**5635**	**5086**	** **	**4438**	** **			** **	** **

The mean individual assignment probabilities (Mean P) with standard deviations for each stock and the corresponding remaining sample sizes are presented for two threshold levels (P > 0.50 and P > 0.59). Increasing the threshold level further decreased the sample sizes of the stocks. The sex ratio (proportion of males) of each stock from the data for P > 0.59, in addition to the prior smolt age distributions used in individual assignments, measured as the proportion of 1- to 2-year-old smolts in the baseline stocks are also given for the studied stocks. In the column “Total”, all individuals were assigned without any threshold.

All of these salmon river stocks originate from the same area, the Gulf of Bothnia in the northern Baltic Sea, and according to DNA microsatellite analysis belong to the same northern genetic Atlantic salmon group within the Baltic Sea [25]. From tagging experiments, it is known that all of these stocks undertake their feeding migration to the southern Baltic Sea or to the Baltic Main Basin, and return back to the Gulf of Bothnia when maturing [26–28]. Fish were sampled only when they were mature and on their spawning migration along the Finnish coast. Catches were pooled as they represent the same spawning migrating fish caught at different sites of the coast. Some migration timing and route differences may still occur and the contribution to the catches may also vary among stocks.

The laboratory analysis of microsatellite variation, the polymerase chain reaction (PCR) and DNA labeling were carried out as described in [22,29,30]. For more details of DNA protocols see [31] https://dx.doi.org/10.17504/protocols.io.bp7ymrpw. Both baseline river samples and offshore catch samples were similarly analyzed. Genetic baseline information was available for 39 Atlantic salmon river stocks from six countries (Sweden, Finland, Russia, Estonia, Latvia and Lithuania) (Fig 1) around the Baltic Sea as previously published baseline data for the analysis [24,29,32] and for catch samples from 2000 to 2013 (2001 was excluded, as no DNA sampling was conducted in that year). For a more detailed explanation of the method, see [24,25,32]. The data were essentially the same as in the study of [19], except for an additional catch sampling year in 2013.

### Genetic stock identification of offshore catch fish data

Multilocus genotype frequencies at 17 DNA microsatellite loci and smolt age distributions of the all 39 potentially contributing baseline river stocks were used to estimate the proportions of separate stocks in the catches with a Bayesian mixture model, BAYES [33] https://archive.fisheries.noaa.gov/afsc/abl/MSA_software.htm. Genetic stock identification, a standard method for offshore salmonid stock composition analysis, has been applied to Atlantic salmon stocks elsewhere as well [34,35].

To use smolt age information in the estimation, the fish in the catch samples were divided into two smolt-age classes according to the smolt age information from scale reading of the catch fish: younger smolts aged 1 to 2 years and older smolts aged 3 to 5 years. As all released hatchery smolts are younger than three years, salmon in the catch samples with a smolt age of three years or greater originated presumably, or *a priori*, from any of the wild stocks, whereas individuals with a smolt age of one or two years may have originated either from a wild or a reared Stock. The use of this information improves the discrimination between wild and reared stocks. Smolt-age distributions have already previously been used as additional data for genetic stock identification [36]. The probability distributions for smolt-age information on the studied stocks are provided in Table 1. The median proportion of young smolts (1–2 years) varied among the wild stocks from 5.6% in wild Tornionjoki fish to 52.2% in Simojoki fish, while it was 100% in all hatchery stocks (Table 1).

For the comparison of fish sizes, we selected from the total archived multi-year catch data for river stocks for which at least 40 individuals were assigned (Table 1). The number of individuals from each of these 14 stocks varied considerably from Öreälven (N = 43) to wild Tornionjoki (N = 1524). The sample sizes from the minor stocks did not allow comparisons in all sex and size classes and they were in those cases omitted.

### Probability of correct individual assignment of catch fish data

For the total dataset, the mean probability of individual assignment of catch fish was 0.79, and for 95% of these assignments, the probability of assignment was between 0.60 and 0.98, when the mean was calculated over the assigned individuals.

For the fish catch size analysis, two threshold levels for the probability of correct individual assignments (IA) of the stock of origin were used: 0.50 and 0.59 (Table 1). For the threshold of 0.59, the total number of individuals of the studied stocks for which information on the sex and sea age was available was 4438, and for the threshold of 0.5, the total number was 5086 individuals (Table 1), while the total dataset comprised 5635 fish. The mean stock-specific assignment probability for the stock of origin varied between 0.78 and 0.96 for the threshold of 0.59, and between 0.73 and 0.95 for the threshold of 0.50 (Table 1). The total number of individuals assigned to each stock varied considerably from 46 to 1524 for common and rare stocks. The threshold was not set higher than 0.59 as this threshold ensures one of the stocks to be clearly the most probable one, and a higher threshold decreases the sample sizes and correspondingly the number of stocks available for comparison. Sample sizes are provided below for each stock in the results. The sex ratio of wild and hatchery-reared catch fish differed markedly. The majority of mature catch fish in the wild stocks were females (proportion of males < 0.5), while the majority in the hatchery stocks were males (proportion of males > 0.5), except among the wild Simojoki salmon, for which the proportion of males was 0.58. In the Simojoki River there have, however, been supplementary hatchery releases.

Testing the percentage of correct individual assignments of the baseline data was not possible with the Bayesian mixture model software so we used the ONCOR software [37,38] to perform leave-one-out tests of 100% of each stock. The 100% tests involve sequentially omitting each fish from the baseline data and then assigning it to the stocks to test the probability of correct assignment of baseline populations. Since ONCOR does not accept non-genetic information, smolt age was omitted from these tests.

### Comparison of methods for estimating stock mean catch length

We compared four genetic stock identification based methods in estimating the size differences of mature salmon stocks on a subset of the real catch data. We used the results of the comparison to select the method to apply to the whole dataset. For the comparison we used data for 2 SW (sea-winter) mature females since it was the largest sex and age group and represented salmon mean size better than 1 SW or 3 SW fish, for which only one sex was available. The true means of the length distributions in sampled matured fish stocks were in this case not known, but we were nonetheless able to compare the differences between estimates, and assess whether the method had any effect on the results. We compared the results for three ways of using individual assignment probabilities (IAPs), in addition to Bayesian mixture modeling (BMM). A Bayesian mixture model directly estimates the length distributions of each stock from the model. For all IAP-based methods, SAS mixed models were applied to the length estimation. Descriptions of the methods are presented below.

#### Method 1

Individual assignment using weighted probabilities or the *direct probability method* (DPM). A theoretically straightforward method is to use posterior source probabilities for individual assignment from mixed-stock analysis directly as weights for the trait of interest for individual fish when calculating the distribution of the trait in the source stock or population. With this method, the whole dataset can be used, and the uncertainty in individual assignments is taken into account at the level it occurs. This can be done using either the maximum assignment probabilities for individuals or the entire matrix of assignment probabilities for all individuals and all potentially contributing baseline stocks.

#### Method 2

Individual assignment with a *threshold method (*THM*) for the minimum assignment probability*. The source of origin of individuals can be determined by using a minimum acceptable threshold level for the probability of correct individual assignment. This method omits the most uncertain individuals from the analysis, and the threshold can be set so that the stock with the highest assignment probability is clearly the most likely stock of origin (e.g. probability of correct stock assignment > 0.5). This THM includes an assumption that the IAs over a certain threshold are correct and are used accordingly, without any uncertainty. This method, however, almost always reduces the original sample size and thus discards potentially useful information. The extent to which this occurs depends on how strictly the threshold is set and the general uncertainty in the data; that is, the level of differentiation (similarity) among the contributing baseline stocks. This method, with a threshold of 0.59, was used by [19] in analyzing differences between salmon stocks in the spawning age distribution, and with a level of 0.8 by Moran et al. [20] in analyzing the occurrence of bacterial kidney disease (BKD).

#### Method 3

A modification of DPM, the *‘reweighting method’ (*RWM*)*. We developed a modification of the DPM (Method 1) that uses a modified (IAP) probability weight, PW = P—(1-P), where P is the maximum posterior source probability that an individual belongs to a baseline stock (IAP). It uses the positive IAP after subtracting the probabilities of all the other stocks when using only the new reweighted probability (PW), which is for that stock alone from the total probability distribution, or the share of the probability distribution that exceeds the level of all other stocks. For a 0.50 versus 0.50 case, the PW is still 0. Any negative weights (i.e. the maximum probability of assignment P is below 0.5) are set to 0, which eliminates individuals with P < 0.5, and the weights for all remaining individuals are normalized to sum to 1. The method is similar to DPM in using weights to estimate the trait distribution for individuals, and because of the reweighting formula, it also in practice applies a threshold with P > 0.5. This RWM reweights the IAPs of P > 0.5, as its weight ranges from 0 to 1, with the original P only ranging from 0.5 to 1. This method avoids the bias caused by individuals assigned to the incorrect stock but includes the uncertainty in correct stock assignments.

#### Method 4

Bayesian mixture modeling (BMM). A fourth method is to use mixture modeling, fitted with either Bayesian or conditional maximum likelihood (CML) methods to directly estimate the distribution of the trait from the catch data, avoiding bias from errors in individual assignments [20,23]. This method has the advantages of using the entire dataset, having high statistical power, and directly estimating the distributions of the trait of interest for individual baseline stocks or defined reporting groups. However, the method requires additional programming and reanalysis of the total dataset, with pooled trait (in our case weight, length, sex, and sea-age) and DNA multilocus genotype data and smolt age data for individuals, since individual assignment (IA) results alone cannot be used.

For the direct Bayesian mixture model test analysis, the data for sea-age at maturity, length, and sex of individual fish were added to the multilocus genotype and smolt age data of each individual, and the length distributions were estimated without the step of individual assignment. This comparison analysis was performed for the length distributions of different sexes of the individual river stocks, but only those fish maturing after two sea years are reported here in the comparison with other methods. The baseline consisted of 39 Atlantic salmon stocks from around the Baltic Sea, and length distributions were estimated for 15 reporting groups, 14 of which were the same most common individual river stocks for which the length distributions are reported, and the 15^th^ was a pooled group of all other stocks.

Similarly to the modeling in Moran et al. [20], we incorporated a model of fish length and sex into the likelihood function of the Bayesian mixture model. Specifically, we modeled the length of fish *m* (*L*_*m*_) as a function of sex (*S*_*m*_, an indicator variable) from reporting group *j* as a normal variate with the mean equal to *β*_0*j*_+*β*_1*j*_*S_m_* and the standard deviation *σ_j_*. The length density for each stock was defined as

$$g(L_{m}|S_{m},j)=\frac{1}{\sigma_{j}\sqrt{2\pi }}e^{-\frac{1}{2\sigma_{j}^{2}}{\left[L_{m}-(\beta_{0j}+\beta_{1j}S_{m})\right]}^{2}}.$$

The likelihood model was defined as

$$L=\prod_{m=1}^{M}\left\{\sum_{j=1}^{n_{s}}\left[g(L_{m}|S_{m},j)\sum_{k=1}^{n_{s^{\prime }(j)}}p_{k(j)}f(X_{m}|Q_{k(j)})\right]\right\},$$

where

*M* = number of mixture individuals,

*n_s_* = number of reporting groups,

*n*_*s*'(*j*)_ = number of stocks in reporting group *j*,

*p*_*k*(*j*)_ = stock proportion *k* of reporting group *j*,

*f*(***X***_*m*_|***Q***_*k*(*j*)_) = relative frequency of genotype and smolt age class ***X***_*m*_ in stock *k* of reporting group *j* given baseline allele frequencies ***Q***_*k*(*j*)_, and *g*(*L_m_*|*S_m_,j*) is defined above.

In addition to the Dirichlet priors used in [33] for ***p*** and ***Q***, we used the standard uninformative prior distribution for a normal regression [39]; Section 14.2): uniform on (***β***_*j*_,log*σ*) or equivalently, $g(\beta_{j},\sigma_{j}^{2}|S_{j})\propto \sigma_{j}^{-2},$ where ***β***_*j*_ = (*β*_0*j*_,*β*_1*j*_) and *σ_j_* are the regression parameters and *S*_*j*_ are the sexes for reporting group *j*. Following [33] ***p*** and ***Q*** were drawn from Dirichlet posterior distributions. Specifying the uniform prior distribution on (***β***_*j*_,log*σ*) provides that the conditional posterior distribution of ***β***_*j*_ given $\sigma_{j}^{2}$ is a normal distribution and that the marginal posterior distribution of $\sigma_{j}^{2}$ has the form of a scaled inverse *χ*^2^ distribution [39]; Section 14.2).

### Calculation example of three IA methods with known means

To present the differences in applying IAPs computed from different methods as weights for the traits of interest, and to gain insights from these methods, we first analyzed a hypothetical example data set of six individuals from two stocks. Individuals for the two stocks were defined to have non-overlapping trait values ([9–11] and [19–21]) and thus known true stock means (10 and 20) so that calculations in the table would be easy to follow. The following three IAP methods were used:

1. THM with the acceptable minimum of P > 0.8 for each individual,
2. DPM with original IAP weights for the trait value for all individuals, and
3. RWM with the positive IAP of the baseline stock with a maximum probability after subtracting the probabilities of all the other stocks. The simple hypothetical example data set consisted of six individuals: 50% from each of two stocks. Individuals were given various assignment probabilities to the stocks, and the true sample mean length in the stocks was set to 10 and 20, so that the direction and magnitude of the deviations from the true means were easy to observe.

### Statistical models for comparing size differences

Differences between river stocks in fish size (weight, length) were analyzed using weighted linear regression analysis. The individual reweighted stock probability (RWM) was used as the weighing factor in the SAS MIXED analysis (SAS Institute, 2012). Pairwise comparisons between rivers were applied using contrasts for all pairs of rivers. Separate models for age groups (1 SW, 2 SW, 3 SW) and sexes were conducted for the year period 2000–2013 (Table 2). All fish from 14 salmon stocks sampled in the years 2000–2013 (2001 excluded) with a known sex, an age of 1–6 sea-winters, and IAP > 0.50 were used in these analyses in cases where the sample size for the analyzed group was sufficient (i.e. N ≥ 10; the sample of 1 SW males from the River Åbyälven analyzed for weight was the smallest, with a sample size N = 11). Interactions were calculated for all models, and the AIC (Akaike information criterion) of the accepted model was checked.

**Table 2:** Models used for analyzing catch size-at-age of Atlantic salmon spawners from different river origin.

Model	Age; sex	N	Response	Predictor (N)	Num DF	Den DF	F-value	P	AIC
***Models for size at age***			*** ***	*** ***	*** ***	*** ***	*** ***	*** ***	
1	1 SW♂	1230	length	stock (11)	10	1219	30.04	<0.001	7680.9
2	2 SW♂	679	length	stock (11)	10	668	6.81	<0.001	4829.5
3	2 SW♀	1919	length	stock (11)	10	1908	20.18	<0.001	12543.6
4	3 SW♀	424	length	stock (10)	9	414	8.57	<0.001	2805.7
5	1 SW♂	1230	weight	stock (11)	10	1219	33.64	<0.001	2628.5
6	2 SW♂	679	weight	stock (11)	10	668	9.60	<0.001	2776.2
7	2 SW♀	1919	weight	stock (11)	10	1908	34.60	<0.001	6786.5
8	3 SW♀	424	weight	stock (10)	9	414	7.00	<0.001	1819.6
**Models for condition factor at age**					** **	** **	** **	** **	
9	2 SW♀	1840	CF	stock (10)	9	1830	32.56	<0.001	-2913.8
10	1 SW♂	1171	CF	stock (10)	9	1161	9.23	<0.001	-2049.0
11	2 SW♀♂	2487	CF	year	1	2483	15.65	<0.093	-3712.0
				wild/reared	1	2483	99.90	<0.001	
				sex	1	2483	49.95	<0.001	

The model number, sample size, age and sex class, sample size (N), response variable, predictor variable, number of stocks included in parentheses, number of degrees of freedom (DF) for the numerator (Num) and denominator (Den), the F value, the probability of significance of the test (P) and Akaike information criterion (AIC) are shown. Interactions between predictors were studied in all models, but they are reported only for the models with the lowest AIC.

The average body weight and length gained by the end of the 1st, 2nd and 3rd sea-winters (1 SW, 2 SW, 3 SW) of each salmon stock were compared by pooling individuals of corresponding stocks and age classes from all catch years, 2000–2013 (2001 excluded). The mean weight and length of the spawners were calculated for all river stocks and for mature males and females separately. Due to differences in age distributions among sexes, only males could be included in the analysis of 1 SW spawners, and only females in the 3 SW analysis. The majority of 1 SW spawners were males (91%, 1 241 males), and there were only 117 mature 1 SW females (2%) in the whole 14-stock data set of 5 635 fish.

The condition factor (CF; the same as the condition index) for each stock was calculated using Fulton’s equation: CF = 1000W/(L^3^) x 100, where W is the weight (kg) and L is the body length (cm) [40]. To apply Fulton’s equation, the slope (b) of the regression equation between log_e_(L) and log_e_(W) should be close to 3.

However, here the samples were only obtained after the first, second or third sea winters, so the regression between weight and length was only based on three points. The average stock-specific condition factor was calculated separately for 2 SW females and 1 SW males (using SAS MEANS procedure).

Differences in condition factors (CF) between the ten most common river stocks were compared with the SAS mixed model in the same way as above for size comparisons. In addition, a separate temporal model for the condition factor of 2 SW salmon was constructed using sex, year, and domestication status (wild or reared) as a predictor in a weighted linear regression. Only individual CF values between 0.70 and 1.30 were included in the analysis, as the relationship between the reported length and weight for the individual fish varied considerably.

## Results

### Baseline accuracy simulation tests

When conducting 100% tests by using each baseline stock in turn as a mixture, both the estimated stock proportions and percentages of correct individual assignments were high. The proportion estimates were very high for most of the stocks: over 0.95 for ten out of fourteen stocks. Values under 0.90 were observed for three stocks, and the lowest was 0.85 for Kalixälven salmon (S1 Table). The percentages of correct individual assignments were all over 90% (S2 Table). Misassignment of individual fishes cannot completely be avoided, but misassigned fish usually have lower probabilities for the stock of origin. In addition, some of the methods used effectively give less weight to salmon with more uncertain assignments. We assume that the uncertainty in individual assignments of the catch fish was relatively correctly estimated, and it also varied somewhat among baseline stocks. With the threshold of P >0.5 the mean probabilities vary between individual stocks from 0.73 to 0.95 and for the threshold of P> 0.59 from 0.78 to 0.93 (Table 1). The true accuracy is somewhat higher as smolt age information was not included here.

### Calculation example of three IA methods with known means

In the test with the six hypothetical example individuals of known lengths, the DPM method showed the poorest performance (Table 3) in using the IAP information as weights. When the true means of the test length distributions were set to 10 and 20, the DPM estimated the means as 12 and 18, respectively. The observed bias not only resulted from the uncertainty of individual assignments, but also from the inclusion of misassigned individuals in the trait estimation, even when their weights were small. This source of uncertainty was eliminated with the threshold method (THM) by leaving out all individuals with a more uncertain assignment to the stock of origin, and using only one source for each individual. The cost of this approach was decreased sample sizes (Table 3) and probable underestimation of the precision, when accepting the assignments over the threshold as known without error (i.e. P = 1). However, the bias was reduced and the estimated means, 9.5 and 19.5, were clearly closer to the true means of 10 and 20 (Table 3), respectively.

**Table 3:** A test example of three ways of using individual assignment probabilities (IAP) to define the mean lengths of two genetic groups (stocks) when the lengths of the individuals were known.

					Probability		1 THM, 0.8		2 DPM		2 DPM		3 Reweighted		RWM		3 RWM	
	True stock		True length		of origin								P-values		Normalized			
Ind.	Origin	Length	Stock1	Stock2	IAP1	IAP2	length1	length2	P1	P2	length1	length2	P1	P2	P1	P2	length1	length2
1	1	9	9		0.9	0.1	9		0.30	0.03	2.70	0.30	0.8	0	0.44	0	4.00	0.00
2	1	10	10		1	0	10		0.33	0.00	3.33	0.00	1	0	0.56	0	5.56	0.00
3	1	11	11		0.5	0.5			0.17	0.17	1.83	1.83	0	0	0.00	0	0.00	0.00
4	2	19		19	0.1	0.9		19	0.03	0.30	0.63	5.70	0	0.8	0	0.44	0.00	8.44
5	2	20		20	0	1		20	0.00	0.33	0.00	6.67	0	1	0	0.56	0.00	11.11
6	2	21		21	0.5	0.5			0.17	0.17	3.50	3.50	0	0	0	0	0.00	0.00
		**MEAN**	**10.0**	**20.0**			**9.50**	**19.50**										
		**SUM**							1	1	**12.0**	**18.0**	1.8	1.8	1	1	**9.56**	**19.56**

The compared methods were (1) the threshold method (THM) with a cut-off probability point of 0.8, (2) the direct use of individual assignment probabilities (DPM) for defining lengths, and (3) reweighting of the original individual assignment probabilities (RWM). For the DPM and RWM methods, the mean of the length distribution in each stock is estimated as a sum of lengths of individuals weighted by IAPs.

The reweighted method (RWM) performed at least as well as the threshold method or even slightly better with estimates of 9.6 and 19.6. The RWM has some advantages over the other threshold methods. It eliminates the most uncertain individuals (i.e. P < 0.5), for which no individual stock has a clear maximum probability, and classifies each individual into only one source stock, which diminishes the bias. In addition, it has the advantage of applying the original uncertainty of the IAP information for individuals with P > 0.5. It thus takes into account the uncertainty of the upper probability distribution, unlike the traditional threshold method. In addition, the method usually includes more individuals in the analysis than the traditional threshold method, since all individuals with an IAP exceeding 0.5 are included with some weight. It also eliminates the problem of deciding on the threshold, as it automatically weights according to the original IAP value for the particular stock or genetic group. Because of these results and the results of the comparisons with the real data, the RWM method was used in the final analysis of growth differences.

### Method comparison with real data

**Fig 2:**
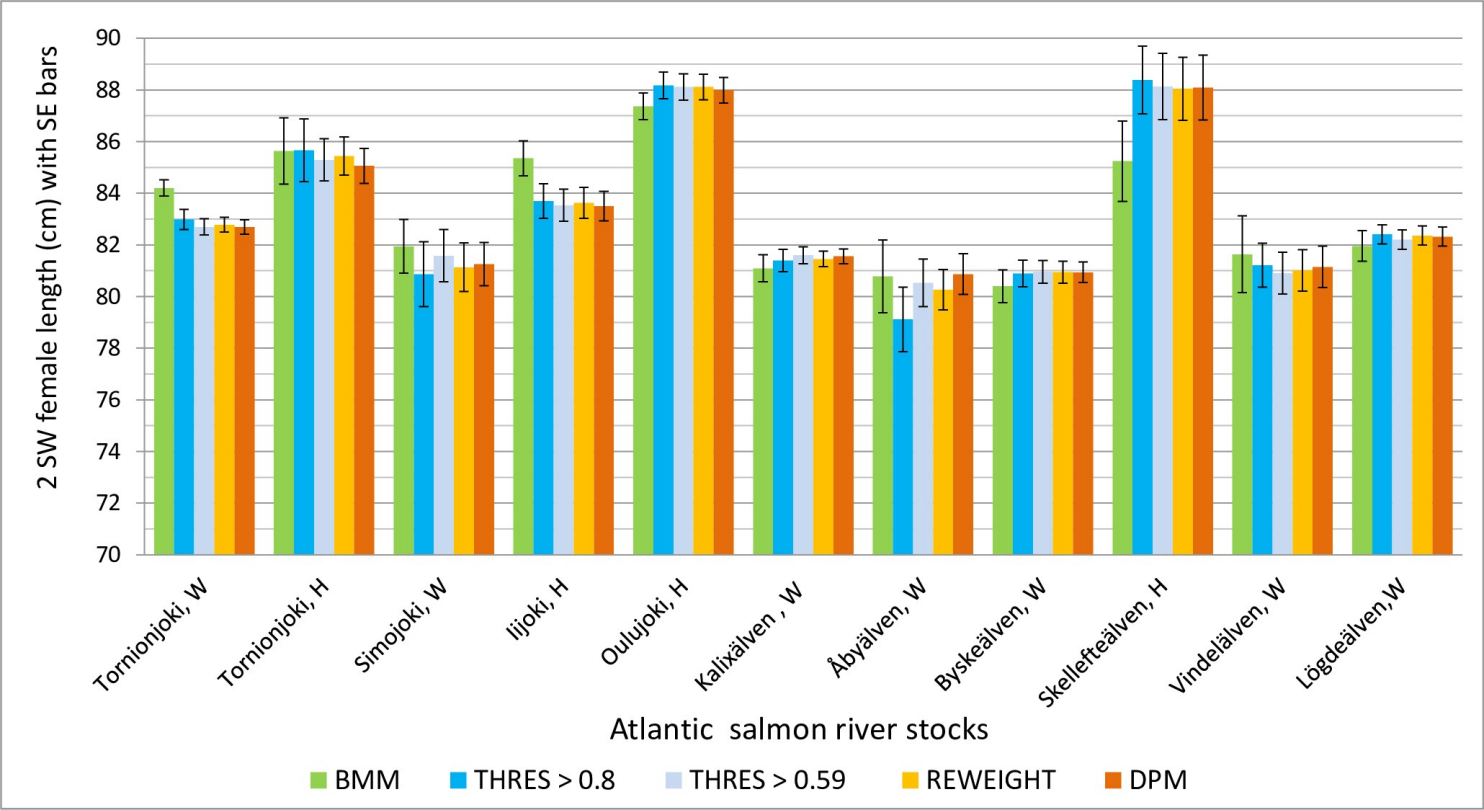
Comparison of methods for estimating the catch length distributions of Atlantic salmon river stocks sampled from the offshore catch. The compared methods were (1) Bayesian mixture modeling (BMM), (2, 3) the threshold methods (THRES) with threshold levels of 0.59 and 0.8, and (4) the reweighting method (REWEIGHT), and (5) the direct probability method (DPM).

When the four GSI methods, namely the direct probability method (DPM), the traditional threshold method (THM) for two probability levels, 0.59 and 0.8, the Bayesian mixture model (BMM) [20,23], and finally the combined threshold probability and reweighting method (RWM) proposed here, were used to estimate the length distributions of 2 SW females from the years 2006 to 2013 (S3 Table, Fig 2), the results were very similar.

In general, the mean catch length estimates for the 2 SW females varied considerably from about 80 cm to 88 cm among the river stocks, but differences between the methods (S3 Table, Fig 2) were not marked. All the methods based on IAPs gave very similar results (Fig 2). Only when the sample size was low (N < 20) with the threshold of 0.8 did more uncertainty occur. The largest differences in the estimates were for the rivers Åbyälven and Skellefteälven, for which only 17 and 18 fish were respectively available when the threshold was set at 0.8 (S3 Table, Fig 2).

The length estimates assessed by the BMM method were more conservative and closer to the overall mean than those of the threshold methods, which had more variation among stocks. It is known that the BMM shrinks the means for the baseline posterior distributions of character frequencies towards the general mean [33]. However, the estimates with all the IAP methods were within the 95% probability intervals of the BMM method in all cases, except for the Tornionjoki wild stock, for which the probability interval was quite narrow, because of the large proportion estimate (0.31) and large sample size (N = 491) (S3 Table). For this stock, all IAP method estimates for the mean length were slightly less (82.7–83.0 cm) than the BMM estimate of 84.2 (83.6–84.8) cm (N = 267–491) (S3 Table). The mean standard error of the estimates increased from 0.6 to 0.8, while the sample size decreased from 1755 to 986, for the IAP-based methods.

In particular, for our data, all threshold methods gave very similar results to the DPM, and the DPM was not noticeably more biased, although it used all individuals in the estimation, as did the BMM method. The uncertainty was understandably larger using the BMM than the threshold methods, in which the most uncertain individuals were excluded.

### Differences in individual mean catch size-at-age among mature salmon river stocks

In the analysis of salmon size differences among river stocks in which the fish origin was genetically identified from the total catch data, the salmon stocks differed significantly (P < 0.001) in mean catch size for both sexes, and in all studied age groups (Table 2). In addition, the pairwise contrast test between stocks demonstrated several significant differences between stock pairs both within and between the wild and reared stock groups in all age classes (S4–S6 Tables)

**Fig 3:**
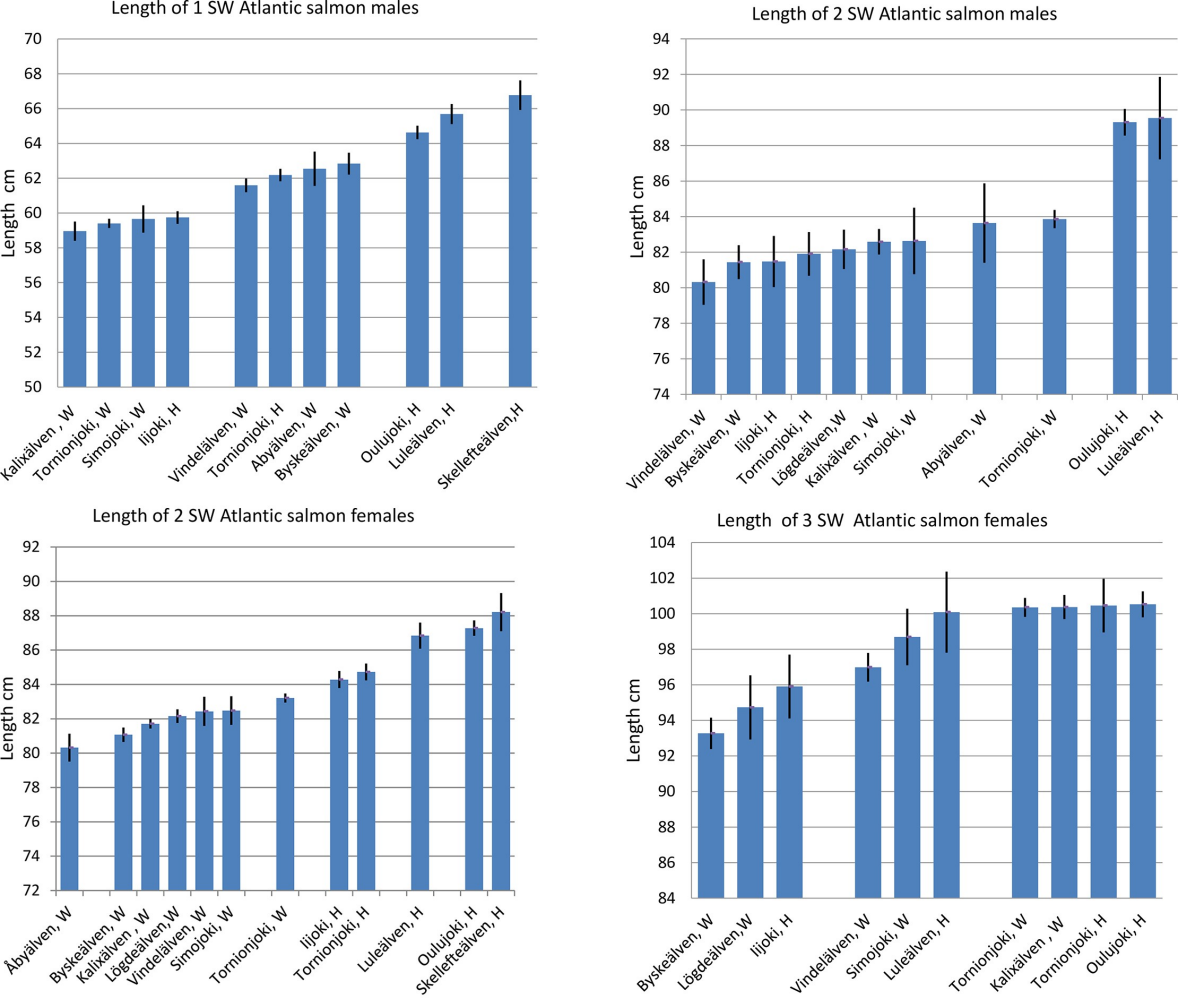
Mean catch length with standard error bars of mature Atlantic salmon aged from 1 to 3 sea-winters in separate river stocks of the Baltic Sea grouped by size classes. A, Mature 1 SW males in catch length classes. B. Mature 2 SW males in catch length classes. C. Mature 2 SW females in catch length classes. D. Mature 3 SW females in catch length classes. There were no statistically significant differences between the smallest and largest means within each size class, but the smallest mean of each size class differed significantly from the smallest mean of the next class. Size classes were based on contrast tests (S4–S6 Tables).

For 1 SW males, the difference between the mean weight of the smallest stock (1.9 kg), with a mean size of less than 60 cm, and the largest stock (Skellefteälven, 2.9 kg), with a mean size of over 66 cm, was as much as a whole kilogram (Tables 4 and 5). Overall, the 1 SW males could be roughly classified into four different length classes, <60, 61–63 cm, 64–66 cm, and >66 cm, when the stocks were organized in order of length and those stocks with no statistically significant differences between the smallest and largest means within each group were included in the same length class. No statistically significant differences occurred between mean lengths of river stocks in the same length class, but the smallest mean of the class differed statistically from the smallest mean of the next class (Fig 3A) (Contrasts S4 Table). The smallest 1 SW males came from the three most northern, wild stocks, the rivers Kalixälven, Tornionjoki, and Simojoki, and in addition from the Iijoki hatchery stock. All the largest males came from the hatchery-reared stocks: Oulujoki, Luleälven, and Skellefteälven, with a mean weight of over 2.5 kg (Table 5, Fig 3A).

**Table 4:** Mean length-at-age and standard deviation (sd) of mature Atlantic salmon river stocks caught from offshore catches in the Baltic Sea area in 2000–2013.

		1 SW ♂	Length cm		2 SW ♂	Length cm/s.28		2 SW ♀	Length cm/s.27		3 SW ♀	Length cm/s.29	
		n	Mean	sd	n	Mean	sd	n	Mean	sd	n	Mean	sd
1	Tornionj., W	265	59.6	3.2	206	83.8	5.6	554	83.2	4.6	144	100.4	5.3
2	Kalixälven	103	58.8	3.8	139	82.6	6.3	406	81.7	4.1	64	100.4	4.0
3	Byskeälven	62	62.4	3.6	59	81.4	6.2	205	81.1	5.0	52	93.3	5.3
4	Vindelälven	27	61.4	1.5	30	80.3	6.3	47	81.4	5.3	36	97.0	5.0
5	Åbyälven	11	62.3	2.3	20	83.6	6.7	46	80.3	3.8	-	-	-
6	Lögdeälven	12	62.6	2.7	38	82.2	6.4	122	82.2	4.2	22	94.7	8.1
7	Simojoki	47	59.6	3.4	24	82.6	6.7	52	82.5	4.7	17	98.7	5.1
** **	**Mean wild**	**527**	**60.1**	**3.4**	**516**	**82.7**	**6.1**	**1432**	**82.2**	**4.5**	**335**	**98.2**	**5.7 **
8	Tornionj., H	224	62.3	4.1	56	81.9	7.3	163	84.7	4.7	25	100.5	5.9
9	Iijoki H	212	59.2	4.3	34	81.5	7.3	132	84.3	4.8	13	95.9	4.7
10	Oulujoki H	171	64.5	4.6	57	89.3	4.9	135	87.3	4.7	38	100.5	4.1
11	Luleälven H	68	65.8	4.2	16	89.5	7.1	57	86.8	4.5	13	100.1	6.7
12	Skelleft., H	40	66.4	4.4	-	-	-	27	88.2	4.9	-	-	-
** **	**Mean hatch.**	**715**	**62.6**	**4.8**	**163**	**85.3**	**7.2**	**514**	**85.8**	**4.9**	**89**	**100.0**	**5.2 **

**Table 5:** Mean weight of mature Atlantic salmon catch fish from different rivers in the Bothnian Sea in 2000–2013.

	1SW ♂	Weight kg		2SW ♂	Weight kg		2SW ♀	Weight kg		3SW ♀	Weight kg	
Stock	n	Mean	sd	n	Mean	sd	n	Mean	sd	n	Mean	sd
Tornionjoki W	265	1.9	0.3	206	5.7	1.2	554	5.5	1.0	144	9.9	1.7
Kalixälven W	103	1.9	0.4	139	5.4	1.3	406	5.0	0.9	64	9.6	1.2
Byskeälven W	62	2.3	0.5	59	5.6	1.4	205	5.4	1.1	52	8.2	1.5
Vindelälven W	27	2.0	0.2	30	5.1	1.4	47	5.6	1.3	36	9.6	1.3
Åbyälven W	11	2.2	0.3	20	6.0	1.3	46	5.1	0.9	-	-	-
Öreälven W	-	-	-	-	-	-	11	5.7	1.0	-	-	-
Lögdeälven W	-	-	-	38	5.5	1.4	122	5.3	1.0	22	8.4	2.0
Simojoki W	47	1.9	0.4	24	5.6	1.4	52	5.4	1.1	17	9.6	1.7
**Mean wild**	**515**	**2.0**	**0.4**	**516**	**5.6**	**1.3**	** 1443**	**5.3**	**1.0**	**335**	**9.4**	**1.7**
Tornionjoki H	224	2.2	0.5	56	5.5	1.5	163	5.9	1.0	25	9.5	1.6
Iijoki H	212	1.9	0.5	34	5.4	1.4	132	5.7	1.0	13	8.8	1.2
Oulujoki H	171	2.6	0.6	57	7.3	1.3	135	6.8	1.2	38	10.5	1.6
Luleälven H	68	2.8	0.7	16	7.5	1.5	57	6.9	0.9	13	10.8	1.9
Skellefteälven H	40	2.9	0.8	15	6.7	1.9	27	7.2	1.3	-	-	-
Ångermanälven H	-	-	-	-	-	1.4	21	7.1	0.9	-	-	-
**Mean hatchery**	**715**	**2.3**	**0.6**	**178**	**6.4**	**1.6**	**528**	**6.3**	**1.2**	**89**	**10.1**	**1.7**
**Sum n**	**1230**			**694**			**1971**			**424**		** **

Wild (W) or reared and sea ranched (H) origin of salmon river stock is indicated.

There were no statistically significant differences between the smallest and largest means within each size class, but the smallest mean of each size class differed significantly from the smallest mean of the next class. Size classes were based on contrast tests (S4–S6 Tables).

The catch weights within both the mature 2 SW male and female groups also varied significantly. The smallest mean for 2 SW males was 5.1 kg (Vildelälven, wild) and the largest was 7.5 kg (Luleälven, hatchery, and 7.3 kg for Oulujoki, hatchery) (Table 5). For 2 SW females, the smallest mean was 5.0 kg (Kalixälven, wild), and the largest was 7.2 kg (Skellefteälven, hatchery).

The length order among the salmon stocks differed somewhat between sexes. The smallest size class for 2 SW males was <82.6 cm, and largest >89 cm (Fig 3B, Table 4). Clearly the smallest 2 SW males came from the river Vindelälven. The largest were the Oulujoki (89.3 cm) and Luleälven (89.5 cm) 2 SW hatchery males, which were on average over 10 cm longer than the smallest 2 SW males.

For 2 SW females, the smallest size class was wild Åbyälven salmon alone with mean size 80.3 cm and the largest was Skellefteälven salmon with mean size 88.2 cm (Fig 3C, Table 4).The mean catch length of the large 3 SW females also varied significantly, from 93.3 cm in Byskeälven (W) to several stocks over 100 cm in Oulujoki (H) (100.5 cm) and Kalixälven (W) (100.4 cm) (Fig 3D) (Table 4). No statistically significant differences were detected between males and females of the same age in their average catch weight or length for 2 SW salmon.

In addition to the weight and length of the caught salmon stocks, their average body condition also differed, as revealed by the differences in the condition factor (S7 Table). The reared and sea-ranched salmon were on average thicker than wild salmon. The reared Oulujoki and Luleälven salmon were on average significantly thicker than those of the other stocks, for both mature 2 SW females (1.00–1.02 vs. 0.90–0.99), and also for mature 1 SW males (S7 Table). The mature 2 SW northern, wild Kalixälven salmon were significantly slimmer than the fish of the other salmon stocks. The condition factor for the reared and wild components of the same Tornionjoki salmon did not differ significantly (S7 Table), so the hatchery fish of this river were very much wild type fish.

The reproduction type (wild or reared) (P < 0.001) and sex (P < 0.001) explained the observed body condition differences, i.e. in the condition factor of the mature 2 SW salmon, while the catch year had no effect (year: P = 0.093) according to the model (Table 2). The small CF was typical for the wild stocks and large CF for the reared stocks (S7 Table).

## Discussion

### Method comparison

In the method comparison with real data, all IAP-based methods (DPM, THM and RWM) gave very similar results of the length distributions of the salmon stocks. The BMM method tended to even out differences between the stocks and was more conservative than all the other methods. This result probably was explained by the fact that the BMM tends to shrink the means for the baseline posterior distributions of character frequencies towards the general mean.

Previously, [20] found poor performance for the DPM in cases where there was a negative correlation between the trait and the genetic distance of the stocks. This can occur when the studied trait has little or no genetic component, or heritability, such as the prevalence of bacterial kidney disease (BKD), the trait they were interested in. In their case, the information from misassigned individuals with low IAPs caused bias in the estimated distributions of the trait, as genetically similar stocks had very different trait distributions. In the DPM, individuals with low assignment probabilities receive more weight than in the THM, in which these individuals are excluded, and the DPM may therefore potentially be more biased. In the test example here, the DPM also showed a tendency to produce biased estimates of the mean of length distributions because of the inclusion of misassigned individuals, even when they had low probabilities.

Moran *et al*., (2014) [20] also compared the performance of the Bayesian mixture model (BMM) with THM by using IAPs from the CML model (ONCOR) [37,38] and by using the maximum a posteriori (MAP) rule with several probability threshold levels (from 0.5 to 0.9), both with simulated and real data. In cases where the traits and genetic distances correlated positively, BMM and THM performed similarly [20]. In cases of negative correlation, BMM performed better, although the classification task was more difficult with negative correlation than with positive correlation for this method as well. In the case of negative correlation, the THM method required higher probability thresholds for sufficient accuracy, to exclude uncertain individuals, which then resulted in lower precision, as sample sizes tended to decrease. In real situations, it is difficult to know how high the threshold should be in order to give optimal results. This problem is avoided with the RWM.

Growth traits such as weight and length usually have high heritability. According to Gjedrem et al. (1988) [41], heritability for 2 SW Atlantic salmon weight was 0.30, for length 0.28, and for growth rate 0.32 [10]. For age at maturity, it has been as high as 0.39 [42]. These traits are thus assumed to correlate positively with general genetic similarity, especially among wild stocks. This also explains the similar results in our case from all methods.

A new potentially useful method was proposed, RWM, which is a modification of DPM. The method weights the trait with the IAPs, but only with the share of probability that exceeds the pooled P of all other stocks. In practice, it thus takes into account only individuals with a higher IAP than 0.5, and the most uncertain individuals are thus omitted. Using genetic individual assignment in general opens up new possibilities for analyzing the distributions of quantitative traits among populations usually occurring in stock mixtures. In addition to salmonids, this could also work for marine fish populations, as long as sufficient genetic differences occur at the DNA marker level.

All of the compared stock assignment methods yielded very similar results for our growth data, and thus indicated that in this case all methods gave sufficiently reliable estimated mean length distributions for individual salmon river stocks from the offshore catch mixture data, and thus confirmed the clear size differences observed among mature salmon stocks of the same age in the same geographical area. The aim of the proposed RWM method was to include observed uncertainty in IAPs by weighting the trait with IAPs and excluding the weights of the most uncertain individuals with a clear rule. In the comparisons here, the RWM performed at least as well as the THM and did not require reanalysis of the data as the BMM did.

### Size differences among caught spawners of Atlantic salmon river stocks

The homing behavior of Atlantic salmon is generally known to lead to genetic differentiation among river stocks, and thus also to the accumulation of adaptive genetic differences [43], for example in growth, which are much more likely to occur in salmonids than in marine fish species.

The growth rates of salmon stocks from different rivers are also known to vary according to the environmental variation resulting from differences in the latitude, geographical type, and size of the home river [44–46]. In some cases, growth differences between wild Atlantic salmon stocks have previously been found [14,47]. Because size-at-age is partly heritable and partly environmentally defined [42,43,48–52], growth differences caused by plain genetic factors are difficult to define in the wild environment. The heritability for age at maturity in the sea was estimated at 0.48 from the offspring—dam regression [53], and it is clearly linked to growth rate [4].

In this study we used catch fish data and the sampled fish had grown in the same sea environment, and size measurements were taken at the same time of the year, for the same life stage, and when the fish had already stopped growing and were mature. Some factors causing variation could thus be excluded and the size of fish was measured in a comparable way. In addition, size-at-age measures were means over 13 years (from 2000 to 2013, excluding 2001), which evens out the effect of annual variation.

The main factors potentially causing differences in the size of mature fish are genetic differences in growth and maturation patterns, and differences in the wild juvenile environments and environmental and genetic effects of hatchery rearing. Here, only the size of the mature component of each salmon stock was measured, while the size of the immature component, which did not return to rivers to spawn but remained on the feeding migration, possibly for at least one additional year, was not measured. In addition there are no data of fish not caught and migration or fishing patterns in the sea may cause the size results to differ from the spawning population sizes, which are the outcome of fish escaping the fishery. Catch size limits or uneven fishery may affect the results of 1 SW salmon. Sample sizes were not sufficient for temporal models for all stocks and sexes. A temporal model was only calculated for the condition factor differences, in which the stocks could be pooled.

The mean final catch size-at-age of the mature salmon clearly differed among river stocks. The size differences were large among the stocks within both the wild and hatchery-reared salmon groups, and especially between the wild and reared stock groups. The mean size of wild salmon spawners was on average smaller than that of the hatchery-reared stocks.

No differences were detected here between the mean catch size of the sexes of the same age, although variation in the size of males was larger than that of females, and the size distribution of the sexes was very different when all ages were included. Because the age distribution of males and females is very different, the final size distribution of the spawners markedly differs, and there is consequently sexual dimorphism in the life cycle of males and females in the spawning population, in which males are mainly 1 and 2 sea-years old and females 2 and 3 sea-years old. This age distribution pattern has also markedly changed since historical times, when it was more even [19]. Some type of sexual dimorphism has also been described, for example, for brown trout (*Salmo trutta* L.) [54].

As the geographical distances between river mouths were not very large, no large climate effect would be expected. Some other life-history traits of salmon, such as smolt age, parr growth, spawning age, and the proportion of repeatedly spawning fish, have been reported to be related to the location and characteristics of the spawning river [16,45,55].

Among hatchery stocks, size differences among mature catch salmon were more pronounced than among the wild stocks. Systematically smaller salmon came from Iijoki and Tornionjoki hatchery stocks, while Oulujoki, Luleälven, and Skellefteälven hatchery stocks had the largest mature salmon (Fig 3). The Iijoki salmon have a very long hatchery rearing history, dating back to the 1960s [56], which may have affected its growth, although its growth in the wild state is unknown. The Tornionjoki hatchery stock does not have a long breeding history, and its broodstocks have been directly established from wild fish. Consequently, there have not been several hatchery generations in its breeding history [8], and it thus has had little time to deviate from the wild component.

The differences in the domestication level among the hatchery stocks may well explain the degree to which hatchery rearing has caused changes in quantitative traits [57,58]. Some broodstocks are directly founded from wild parr, such as the Tornionjoki hatchery stock, and some from sea-ranched spawners, including several Swedish hatchery stocks, and some broodstocks have already experienced several successive generations in the hatchery, such as the Iijoki and Oulujoki salmon stocks [56]. Both large growing stocks, Oulujoki and Skellefteälven, are only based on hatchery production.

Both females and males in the hatchery-reared stocks were on average larger than in the wild stocks for all sea-age classes, which suggest that selective factors and unintentional selection in hatchery rearing in general have favored faster growth. Similar trends have also been observed in previous studies on Baltic salmon [14,15,57], and the same has also been reported elsewhere [59–61]. The smolt size of hatchery fish may be larger than that of wild fish, but hatchery rearing is also known to favor faster growth.

There is considerable evidence that hatchery-reared salmon, and sea-ranched salmon to some extent, differ from wild salmon in many important fitness-related quantitative traits [61–63]. In addition to hatchery selection and domestication, the juvenile rearing environment may affect these growth differences, and hatchery-reared smolts are usually already larger than wild ones at the beginning of the sea migration [14,64].

The previous observations of larger size-at-age of hatchery-reared catch fish agrees well with our current findings of size differences among Baltic salmon stocks, and the results with the current method are in this respect also consistent with those of the previous studies. Here, we were able to obtain detailed information on the stock-level catch size-at-age differences and for separate sexes, in addition to the differences between wild and reared stock groups. The factors underlying the differences, such as the combination of growth and environmental factors, as well as juvenile environment variables, or potential uneven migration or fishery, require further research. This type of catch size analysis, however, also enables further investigation of temporal variation and the combination of other potentially affecting factors, when enough numbers of individuals are available.

## Supporting information

S1 Table Proportion of correct estimation of the 100% contribution of each baseline stock in turn. (DOCX)

S2 Table Percentage of correct individual assignments of baseline stock individuals in a 100% test, in which each baseline salmon stock alone was used in turn as mixed sample. (DOCX)

S3 Table Comparison of five methods for assessing the mean length distributions of 2 SW females in the catch of Atlantic salmon from eleven river stocks over the years 2006 to 2013 in the Baltic Sea. (DOCX)

S4 Table The significance of pairwise catch length and weight differences between river stocks for 1 SW Atlantic salmon in the Baltic Sea. (DOCX)

S5 Table The significance of pairwise catch length differences between Atlantic salmon river stocks for 2SW females and males, separately in the Baltic Sea. (DOCX)

S6 Table Significance of pairwise weight and length differences between river stocks for 3 SW female Atlantic salmon in the Baltic Sea. (DOCX)

S7 Table The significance of pairwise condition factor (CF) differences between Atlantic salmon river stocks and CF means for the 2 SW females and 1 SW males. (DOCX)
