# Supplementary material for: Large inter-stock differences in catch size-at-age of mature Atlantic salmon observed by using genetic individual origin assignment from catch data
Source: PLoS One. 2021 Apr 6;16(4):e0247435. doi: 10.1371/journal.pone.0247435 (PMC8023481; doi:10.1371/journal.pone.0247435)
Supplement: S1 Table — (DOCX) [file pone.0247435.s001.docx]

**S1 Table.** **Proportion of correct estimation of the 100% contribution of each baseline stock in turn.**

|  | **N** | **Stock** | **Mean** | **sd** | **2.50 %** | **Median** | **97.50 %** |
| --- | --- | --- | --- | --- | --- | --- | --- |
| 1 | 210 | Tornionjoki W | 0.89 | 0.04 | 0.82 | 0.89 | 0.96 |
| 2 | 187 | Tornionjoki H | 0.99 | 0.01 | 0.98 | 1.00 | 1.00 |
| 3 | 174 | Simojoki | 0.91 | 0.02 | 0.86 | 0.91 | 0.96 |
| 4 | 179 | Iijoki | 0.99 | 0.01 | 0.96 | 0.99 | 1.00 |
| 5 | 135 | Oulujoki | 0.89 | 0.03 | 0.82 | 0.89 | 0.94 |
| 6 | 200 | Kalixälven | 0.85 | 0.04 | 0.76 | 0.85 | 0.93 |
| 7 | 90 | Luleälven | 0.99 | 0.01 | 0.96 | 0.99 | 1.00 |
| 8 | 102 | Åbyälven | 0.98 | 0.02 | 0.95 | 0.99 | 1.00 |
| 9 | 105 | Byskeälven | 0.98 | 0.02 | 0.94 | 0.99 | 1.00 |
| 10 | 58 | Skellefteälven | 0.98 | 0.02 | 0.93 | 0.99 | 1.00 |
| 11 | 149 | Vindelälven | 0.99 | 0.01 | 0.97 | 1.00 | 1.00 |
| 12 | 54 | Öreälven | 0.96 | 0.03 | 0.88 | 0.97 | 1.00 |
| 13 | 102 | Lögdeälven | 0.99 | 0.01 | 0.95 | 0.99 | 1.00 |
| 14 | 79 | Ångermanälven | 0.99 | 0.01 | 0.96 | 0.99 | 1.00 |
|  | 1824 |  | 0.96 |  |  |  |  |
