# Supplementary material for: Large inter-stock differences in catch size-at-age of mature Atlantic salmon observed by using genetic individual origin assignment from catch data
Source: PLoS One. 2021 Apr 6;16(4):e0247435. doi: 10.1371/journal.pone.0247435 (PMC8023481; doi:10.1371/journal.pone.0247435)
Supplement: S2 Table — (DOCX) [file pone.0247435.s002.docx]

**S2 Table. Percentage of correct individual assignments of baseline stock individuals in a 100% test, in which each baseline salmon stock alone was used in turn as mixed sample.**

|  | **Salmon river stock** | **N** | **% Correct** | **Mean P** | **Largest misidentification** | |
| --- | --- | --- | --- | --- | --- | --- |
| 1 | Tornionjoki W | 210 | 94.29 % | 0.93 | Kalixälven | 5.71% |
| 2 | Tornionjoki H | 187 | 100.00 % | 1.00 | - | - |
| 3 | Simojoki | 174 | 93.10 % | 0.98 | Kalixälven | 5.75% |
| 4 | Iijoki | 179 | 100.00 % | 0.99 | - | - |
| 5 | Oulujoki | 135 | 90.37 % | 0.98 | Iijoki | 8.89% |
| 6 | Kalixälven | 200 | 90.50 % | 0.91 | Tornionjoki W | 9.50% |
| 7 | Luleälven | 90 | 100.00 % | 1.00 | - | - |
| 8 | Åbyälven | 101 | 100.00 % | 1.00 | - | - |
| 9 | Byskeälven | 105 | 100.00 % | 1.00 | - | - |
| 10 | Skellefteälven | 57 | 100.00 % | 1.00 | - | - |
| 11 | Vindelälven | 149 | 100.00 % | 1.00 | - | - |
| 12 | Öreälven | 54 | 98.15 % | 0.99 | Lögdeälven | 1.85% |
| 13 | Lögdeälven | 91 | 100.00 % | 1.00 | - | - |
| 14 | Ångermanälven | 77 | 100.00 % | 1.00 | - | - |

The river stock, sample size, percentage of correctly assigned individuals, mean probability of the correctly assigned individuals, river stock of most common misassignments, and its percentage are given.
