# Supplementary material for: Large inter-stock differences in catch size-at-age of mature Atlantic salmon observed by using genetic individual origin assignment from catch data
Source: PLoS One. 2021 Apr 6;16(4):e0247435. doi: 10.1371/journal.pone.0247435 (PMC8023481; doi:10.1371/journal.pone.0247435)
Supplement: S3 Table — (DOCX) [file pone.0247435.s003.docx]

**S3 Table**. **Comparison of five methods for assessing the mean length distributions of 2 SW females in the catch of Atlantic salmon from eleven river stocks over the years 2006 to 2013 in the Baltic Sea.**

|  | **Stock** | **Tornionjoki. W** | **Tornionjoki. H** | **Simojoki. W** | **Iijoki. H** | **Oulujoki. H** | **Kalixälven . W** | **Abyälven. W** | **Byskeälven. W** | **Skellefteälven.H** | **Vindelälven. W** | **Lögdeälven.W** | ***Mean/Total N*** |
| --- | --- | --- | --- | --- | --- | --- | --- | --- | --- | --- | --- | --- | --- |
| **1 DPM** | | | |  |  |  |  |  |  |  |  |  |  |
|  | **Mean** | **82.7** | **85.1** | **81.3** | **83.5** | **88.0** | **81.6** | **80.9** | **80.9** | **88.1** | **81.2** | **82.3** |  |
|  | sd | 5.7 | 5.6 | 5.1 | 5.6 | 4.6 | 5.0 | 4.9 | 5.4 | 5.7 | 5.8 | 4.0 | *5.2* |
|  | se | 0.3 | 0.7 | 0.8 | 0.6 | 0.5 | 0.3 | 0.8 | 0.4 | 1.3 | 0.8 | 0.4 | *0.6* |
|  | Med. | 83 | 85 | 80 | 84 | 88 | 81.5 | 81 | 81 | 90 | 81 | 82 |  |
|  | *N* | *532* | *95* | *49* | *117* | *95* | *379* | *55* | *229* | *23* | *61* | *120* | *1755* |
| **2 BMM** | | | | |  |  |  |  |  |  |  |  |  |
|  | **Mean** | **84.2** | **85.6** | **81.9** | **85.4** | **87.4** | **81.1** | **80.8** | **80.4** | **85.2** | **81.6** | **82.0** |  |
|  | se | 0.3 | 1.3 | 1.0 | 0.7 | 0.5 | 0.5 | 1.4 | 0.6 | 1.6 | 1.5 | 0.6 | *0.9* |
|  | 95% PI | 83.6 | 83.2 | 79.9 | 84 | 86.3 | 80.1 | 78 | 79.1 | 82.2 | 78.7 | 80.8 |  |
|  | 95% PI | 84.8 | 88.3 | 84 | 86.7 | 88.4 | 82.1 | 83.6 | 81.7 | 88.3 | 84.5 | 83.1 |  |
|  | Prop. | 0.31 | 0.03 | 0.03 | 0.07 | 0.06 | 0.16 | 0.03 | 0.09 | 0.02 | 0.02 | 0.06 | *2378* |
| **3 RWM > 0.50** | | | |  |  |  |  |  |  |  |  |  |  |
|  | **Mean** | **82.8** | **85.4** | **81.1** | **83.6** | **88.1** | **81.5** | **80.3** | **80.9** | **88.0** | **81.0** | **82.4** |  |
|  | sd | 5.0 | 4.8 | 4.7 | 5.3 | 4.5 | 4.2 | 3.7 | 5.1 | 5.3 | 5.4 | 3.9 | *4.7* |
|  | se | 0.3 | 0.7 | 0.9 | 0.6 | 0.5 | 0.3 | 0.8 | 0.4 | 1.2 | 0.8 | 0.4 | *0.6* |
|  | Med. | 83 | 85 | 80 | 84 | 88 | 81 | 80 | 81 | 90 | 81 | 82 |  |
|  | *N* | *491* | *80* | *40* | *106* | *90* | *359* | *48* | *209* | *23* | *53* | *119* | *1618* |
| **4 THM > 0.59** | | |  |  |  |  |  |  |  |  |  |  |  |
|  | **Mean** | **82.7** | **85.3** | **81.6** | **83.5** | **88.1** | **81.6** | **80.5** | **81.0** | **88.1** | **80.9** | **82.2** |  |
|  | sd | 6.5 | 6.6 | 5.6 | 6.2 | 4.8 | 5.7 | 5.7 | 6.1 | 6.1 | 5.8 | 4.1 | *5.7* |
|  | se | 0.3 | 0.8 | 1.0 | 0.6 | 0.5 | 0.3 | 0.9 | 0.4 | 1.3 | 0.8 | 0.4 | *0.7* |
|  | Med. | 83 | 85 | 80 | 84 | 88 | 82 | 81 | 81 | 90 | 81 | 82 |  |
|  | *N* | *429* | *65* | *31* | *101* | *88* | *302* | *38* | *192* | *23* | *51* | *118* | *1438* |
| **5 THM > 0.80** | | |  |  |  |  |  |  |  |  |  |  |  |
|  | **Mean** | **83.0** | **85.7** | **80.9** | **83.7** | **88.2** | **81.4** | **79.1** | **80.9** | **88.4** | **81.2** | **82.4** |  |
|  | sd | 6.4 | 7.3 | 5.9 | 5.9 | 4.7 | 5.7 | 5.2 | 6.0 | 5.6 | 5.8 | 4.0 | *5.7* |
|  | se | 0.4 | 1.2 | 1.3 | 0.7 | 0.5 | 0.4 | 1.3 | 0.5 | 1.3 | 0.9 | 0.4 | *0.8* |
|  | Med. | 83 | 85 | 80 | 84 | 88 | 81 | 80 | 81 | 90 | 81 | 82 |  |
|  | *N* | *267* | *36* | *22* | *76* | *82* | *170* | *17* | *139* | *18* | *46* | *113* | *986* |

The means, standard deviations (sd), standard errors (se) and medians (Med.) for the length distributions are given: (1) Direct individual assignment probabilities (DPM); (2) Bayesian mixture modeling (BMM) without individual assignment data; (3) reweighted IAP probabilities (RWM); (4) IAP with a threshold value (THM) of 0.59 for the individual assignment probability, and (5) threshold of 0.8 as a cut-off for the individual assignment probability (THM).
