# Supplementary material for: Large inter-stock differences in catch size-at-age of mature Atlantic salmon observed by using genetic individual origin assignment from catch data
Source: PLoS One. 2021 Apr 6;16(4):e0247435. doi: 10.1371/journal.pone.0247435 (PMC8023481; doi:10.1371/journal.pone.0247435)
Supplement: S4 Table — (DOCX) [file pone.0247435.s004.docx]

**S4 Table. The significance of pairwise catch length and weight differences between river stocks for 1 SW Atlantic salmon in the Baltic Sea.**

| ***Weight***  ***1 SW*♂**  ***Length***  ***1 SW* ♂** | **Tornionjoki W** | **Kalixälven W** | | **Byskeälven W** | | **Vindelälven W** | | **Åbyälven W** | | **Simojoki W** | **Tornionjoki H** | **Iijoki H** | **Oulujoki H** | **Luleälven H** | **Skellefteälven H** |
| --- | --- | --- | --- | --- | --- | --- | --- | --- | --- | --- | --- | --- | --- | --- | --- |
| **Tornionjoki W** |  | | ns | | *** | | ns | | ns | ns | *** | ns | *** | *** | *** |
| **Kalixälven W** | ns | |  | | *** | | ns | | ns | ns | *** | ns | *** | *** | *** |
| **Byskeälven W** | *** | | *** | |  | | ns | | ns | *** | ns | *** | ** | *** | *** |
| **Vindelälven W** | ns | | * | | ns | |  | | ns | ns | ns | ns | *** | *** | *** |
| **Åbyälven W** | ns | | * | | ns | | ns | |  | ns | ns | ns | ns | * | ** |
| **Simojoki W** | ns | | ns | | ** | | ns | | ns |  | ** | ns | *** | *** | *** |
| **Tornionjoki H** | *** | | *** | | ns | | ns | | ns | ** |  | *** | *** | *** | *** |
| **Iijoki H** | ns | | ns | | *** | | * | | ns | ns | *** |  | *** | *** | *** |
| **Oulujoki H** | *** | | *** | | ** | | *** | | ns | *** | *** | *** |  | * | ** |
| **Luleälven H** | *** | | *** | | *** | | *** | | * | *** | *** | *** | ns |  | ns |
| **Skellefteälven H** | *** | | *** | | *** | | *** | | * | *** | *** | *** | * | ns |  |
| ***n*** | *265* | | *103* | | *62* | | *27* | | *11* | *47* | *224* | *212* | *171* | *68* | *40* |
| **Mean (kg)** | **1.9** | | **1.9** | | **2.3** | | **2.0** | | **2.2** | **1.9** | **2.2** | **1.9** | **2.6** | **2.8** | **2.9** |
| **sd (kg)** | 0.3 | | 0.4 | | 0.5 | | 0.2 | | 0.3 | 0.4 | 0.5 | 0.5 | 0.6 | 0.7 | 0.8 |
| ***n*** | *265* | | *103* | | *62* | | *27* | | *11* | *47* | *224* | *212* | *171* | *68* | *40* |
| **Mean (cm)** | **59.6** | | **58.8** | | **62.4** | | **61.4** | | **62.3** | **59.6** | **62.3** | **59.2** | **64.5** | **65.8** | **66.4** |
| **sd (cm)** | 3.2 | | 3.8 | | 3.6 | | 1.5 | | 2.3 | 3.4 | 4.1 | 4.3 | 4.6 | 4.2 | 4.4 |

For each stock the mean weight and length with standard deviations (sd) are shown.
