# Supplementary material for: Large inter-stock differences in catch size-at-age of mature Atlantic salmon observed by using genetic individual origin assignment from catch data
Source: PLoS One. 2021 Apr 6;16(4):e0247435. doi: 10.1371/journal.pone.0247435 (PMC8023481; doi:10.1371/journal.pone.0247435)
Supplement: S5 Table — (DOCX) [file pone.0247435.s005.docx]

**S5 Table. The significance of pairwise catch length differences between Atlantic salmon river stocks for 2SW females and males, separately in the Baltic Sea.**

| ***2 SW ♀.***  ***Length diff.***  ***2 SW* ♂**  ***Length diff*.** | **Tornionjoki W** | **Kalixälven W** | **Byskeälven W** | **Vindelälven W** | **Åbyälven W** | **Lögdeälven W** | **Simojoki W** | **Tornionjoki H** | **Iijoki H** | **Oulujoki H** | **Luleälven H** |
| --- | --- | --- | --- | --- | --- | --- | --- | --- | --- | --- | --- |
| **Tornionjoki W** |  | *** | *** | ns | *** | * | ns | ** | * | *** | ** |
| **Kalixälven W** | ns |  | ns | ns | *** | ns | ns | *** | *** | *** | ns |
| **Byskeälven W** | * | ns |  | ns | *** | ns | ns | *** | *** | *** | ns |
| **Vindelälven W** | ** | ns | ns |  | *** | ns | ns | ** | * | *** | ns |
| **Åbyälven W** | ** | ** | *** | *** |  | *** | *** | * | ** | ns | *** |
| **Lögdeälven W** | ns | ns | ns | ns | ** |  | ns | *** | *** | *** | ns |
| **Simojoki W** | ns | ns | ns | ns | ** | ns |  | * | ns | *** | ns |
| **Tornionjoki H** | ns | ns | ns | ns | *** | ns | ns |  | ns | *** | *** |
| **Iijoki H** | ns | ns | ns | ns | *** | ns | ns | ns |  | *** | *** |
| **Oulujoki H** | *** | *** | *** | *** | ns | *** | *** | *** | *** |  | *** |
| **Luleälven H** | ns | ns | ns | ns | * | ns | ns | ns | ns | * |  |
| ***n* ♀** | *554* | *406* | *205* | *47* | *46* | *122* | *52* | *163* | *132* | *135* | *57* |
| ***n ♂*** | *206* | *139* | *59* | *30* | *20* | *38* | *24* | *56* | *34* | *57* | *16* |

The significance of the length differences between pairs of mature 2 SW Atlantic salmon female stocks from different rivers is shown in the upper triangle and that of the 2 SW male stocks in the lower triangle. Sample sizes (n) for each stock are given. The wild (W) and hatchery (H) origin of each stock is indicated.
