# Supplementary material for: Large inter-stock differences in catch size-at-age of mature Atlantic salmon observed by using genetic individual origin assignment from catch data
Source: PLoS One. 2021 Apr 6;16(4):e0247435. doi: 10.1371/journal.pone.0247435 (PMC8023481; doi:10.1371/journal.pone.0247435)
Supplement: S6 Table — (DOCX) [file pone.0247435.s006.docx]

**S6 Table. Significance of pairwise weight and length differences between river stocks for 3 SW female Atlantic salmon in the Baltic Sea.**

| **Weight**  **3 SW♀**  **Length**  **3 SW ♀** | **Tornionjoki W** | **Kalixälven W** | **Byskeälven W** | **Vindelälven W** | **Lögdeälven W** | **Simojoki W** | **Tornionjoki H** | **Iijoki H** | **Oulujoki H** | **Luleälven H** |
| --- | --- | --- | --- | --- | --- | --- | --- | --- | --- | --- |
| **Tornionjoki W** |  | ns | *** | ns | *** | ns | ns | ns | ns | ns |
| **Kalixälven W** | ns |  | *** | ns | ** | ns | ns | ns | * | * |
| **Byskeälven W** | *** | *** |  | *** | ns | * | ** | ns | *** | *** |
| **Vindelälven W** | ** | ** | ** |  | ** | ns | ns | ns | * | * |
| **Lögdeälven W** | *** | *** | ns | ns |  | * | * | ns | *** | *** |
| **Simojoki W** | ns | ns | ** | ns | * |  | ns | ns | ns | ns |
| **Tornionjoki H** | ns | ns | *** | * | ** | ns |  | ns | * | * |
| **Iijoki H** | * | * | ns | ns | ns | ns | ns |  | * | * |
| **Oulujoki H** | ns | ns | *** | ** | *** | ns | ns | * |  | ns |
| **Luleälven H** | ns | ns | *** | ns | * | ns | ns | ns | ns |  |
| ***n*** | *144* | *64* | *52* | *36* | *22* | *17* | *25* | *13* | *38* | *13* |
| **Mean (kg)** | 9.9 | 9.6 | 8.2 | 9.6 | 8.4 | 9.6 | 9.5 | 8.8 | 10.5 | 10.8 |
| **sd (kg)** | 1.7 | 1.2 | 1.5 | 1.3 | 2.0 | 1.7 | 1.6 | 1.2 | 1.6 | 1.9 |
| **n** | 144 | 64 | 52 | 36 | 22 | 17 | 25 | 13 | 38 | 13 |
| **Mean (cm)** | 100.4 | 100.4 | 93.3 | 97.0 | 94.7 | 98.7 | 100.5 | 96.0 | 100.5 | 100.1 |
| **sd (cm)** | 5.3 | 4.0 | 5.3 | 4.5 | 8.1 | 5.1 | 5.9 | 4.7 | 4.1 | 6.7 |

Sample sizes (*n*) for each stock and the mean weight and length with standard deviations (sd) are also shown.
