# Supplementary material for: Large inter-stock differences in catch size-at-age of mature Atlantic salmon observed by using genetic individual origin assignment from catch data
Source: PLoS One. 2021 Apr 6;16(4):e0247435. doi: 10.1371/journal.pone.0247435 (PMC8023481; doi:10.1371/journal.pone.0247435)
Supplement: S7 Table — (DOCX) [file pone.0247435.s007.docx]

**S7 Table. The significance of pairwise condition factor (CF) differences between Atlantic salmon river stocks and CF means for the 2 SW females and 1 SW males.**

| **CF 2 SW**  **females**  **CF**  **1 SW males** | **Tornionjoki W** | **Kalixälven W** | **Byskeälven W** | **Vindelälven W** | **Lögdeälven W** | **Simojoki W** | **Tornionjoki H** | **Iijoki H** | **Oulujoki H** | **Luleälven H** |
| --- | --- | --- | --- | --- | --- | --- | --- | --- | --- | --- |
| **Tornionjoki W** |  | *** | *** | *** | ns | ns | ns | ns | *** | *** |
| **Kalixälven W** | ns |  | *** | *** | *** | *** | *** | *** | *** | *** |
| **Byskeälven W** | *** | ** |  | ns | *** | * | *** | *** | ** | *** |
| **Vindelälven W** | ns | ns | ** |  | *** | * | ** | *** | ns | ** |
| **Lögdeälven W** | ns | ns | ns | ns |  | ns | ns | ns | *** | *** |
| **Simojoki W** | ns | ns | ** | ns | ns |  | ns | ns | *** | *** |
| **Tornionjoki H** | ns | ns | * | ns | ns | ns |  | ns | *** | *** |
| **Iijoki H** | ** | * | ns | * | ns | * | ns |  | *** | *** |
| **Oulujoki H** | *** | *** | ns | *** | ns | *** | *** | *** |  | ns |
| **Luleälven H** | *** | *** | ns | *** | ns | *** | *** | ** | ns |  |
| **2 SW females n** | **538** | **401** | **199** | **47** | **122** | **52** | **161** | **132** | **132** | **56** |
| **Mean CF** | 0.93 | 0.90 | 0.99 | 0.99 | 0.94 | 0.96 | 0.94 | 0.94 | 1.02 | 1.04 |
| **sd CF** | 0.07 | 0.07 | 0.09 | 0.10 | 0.08 | 0.08 | 0.07 | 0.07 | 0.08 | 0.08 |
| **1 SW males n** | **259** | **100** | **62** | **27** | **12** | **46** | **218** | **209** | **171** | **67** |
| **Mean CF** | 0.89 | 0.88 | 0.93 | 0.88 | 0.92 | 0.88 | 0.90 | 0.91 | 0.94 | 0.95 |
| **sd CF** | 0.07 | 0.06 | 0.07 | 0.06 | 0.08 | 0.07 | 0.06 | 0.07 | 0.08 | 0.09 |

The significance of pairwise condition factor differences between mature 2 SW female Atlantic salmon stocks are shown in the upper triangle of the table and that of the mature 1 SW males in the lower triangle. The mean condition factor (CF) of each stock and its standard deviation (sd), and sample sizes (n) in the stocks are given.
